# The SARC-F score alone may underdetect sarcopaenia risk in primary care: The case for combined functional assessment and rehabilitation referral

**DOI:** 10.51866/lte.26-00047

**Published:** 2026-10-03

**Authors:** Ebru Kübra Taşpolat

**Affiliations:** 1 Department of Physical Medicine and Rehabilitation, Elbistan State Hospital, Kahramanmaraş-Turkey.

**Keywords:** Sarcopaenia, Primary health care, Mass screening, Muscle strength, Rehabilitation

**Dear Editor,**

I read with interest the recent article by Tay et al., titled ‘SARC-F score compared with hand grip strength for screening possible sarcopaenia among community-dwelling older adults: A multicentre cross-sectional study in Malaysia’.^1^ This multi-centre study provides valuable real-world evidence on the performance of a commonly recommended screening tool in Malaysian primary care. From a physical medicine and rehabilitation perspective, several findings have implications for both case finding and subsequent management.

First, the sensitivity of the SARC-F score at the conventional cut-off of >4 was only 34.7%, despite a specificity of 87.5%, and its agreement with hand grip strength (HGS)-based classification was poor to fair (**k**=0.219).^1^ Propst et al. reported a similar pattern and proposed a lower cut-off of >2,^2^ and taken together, these findings support the authors’ own conclusion that the SARC-F score alone is not adequate for case finding. I do not wish to labour this point further; instead, I would like to draw attention to two areas the present study does not address: the Asian Working Group for Sarcopenia (AWGS) 2025 update to the diagnostic framework and what should happen clinically once a patient screens positive.

Second, the study used the AWGS 2019 criteria.^3^ Since the data were collected, the AWGS 2025 consensus has been published, and this changes the picture in a few respects worth noting. The focus has broadened from sarcopaenia as a discrete diagnosis to muscle health more generally; case finding now extends down to age of 50 years rather than starting in older age; the diagnostic algorithm has been simplified; and management is framed explicitly around combining resistance exercise with nutritional supplementation.^4^ For Malaysian primary care practitioners, this matters in practice: A simpler algorithm is easier to apply during a routine consultation, and starting earlier leaves more room to intervene before decline sets in. Separately, Barbosa-Silva et al. found that adding calf circumference to the SARC-F score (SARC-CalF) improves sensitivity without much loss of specificity, and this approach adds almost no time to a visit.^5^ Future Malaysian studies should compare the SARC-F score, the SARC-CalF and the AWGS 2025 framework, since lowering the SARC-F threshold is not the only way, and may not be the best way, to address underdetection.

Third, screening on its own achieves little unless it is tied to a clear next step, and this is where I think the discussion is most needed. A positive SARC-F result, or a negative result in a patient who still looks clinically frail, should prompt objective testing: HGS measurement, gait speed test, the chair stand test or the Short Physical Performance Battery. Where weakness or functional impairment is confirmed, patients should be referred for a rehabilitation programme built around progressive resistance training and nutritional support, in line with the AWGS 2025 recommendations.^4^ Patients with other markers of frailty are worth testing objectively regardless of their SARC-F score, since a negative screen can be falsely reassuring in this group. None of this requires new infrastructure, only a protocol that primary care clinics can follow consistently.

Finally, as the authors noted, the cross-sectional design cannot indicate whether earlier detection actually changes outcomes. A longitudinal study examining whether this kind of pathway — screening, objective assessment and timely rehabilitation — reduces falls, hospitalisations or functional decline would be a natural next step and one that would matter directly for how primary care resources are planned.

I congratulate the authors on a useful and timely study and hope these comments, particularly on the AWGS 2025 update and on what should follow a positive screen, are useful as this area moves forward in Malaysian primary care.
